# 1-Benzoyl-3,3-dinitro­azetidine

**DOI:** 10.1107/S1600536809051290

**Published:** 2009-12-04

**Authors:** Biao Yan, Hai-Xia Ma, Jun-Feng Li, Yu-Lei Guan, Ji-Rong Song

**Affiliations:** a School of Chemistry and Chemical Engineering, Yulin University, Yulin 719000 Shaanxi, People’s Republic of China; b School of Chemical Engineering, Northwest University, Xi’an 710069 Shaanxi, People’s Republic of China

## Abstract

In the title *gem*-dinitro­azetidine derivative, C_10_H_9_N_3_O_5_, the azetidine ring is almost planar, the maximum value of the endocyclic torsion angle being 0.92 (14)°. The gem-dinitro groups are mutually perpendicular and the dihedral angle between the azetidine and benzene rings is 46.70 (10)°

## Related literature

For energetic materials based on 3,3-dinitro­azetidine, see: Archibald *et al.* (1990 ▶); Gao *et al.* (2009 ▶); Hiskey & Coburn (1994*a* ▶,*b* ▶); Ma, Yan, Li, Guan *et al.* (2009 ▶); Ma, Yan, Li, Song & Hu (2009 ▶); Ma, Yan, Song *et al.* (2009 ▶).
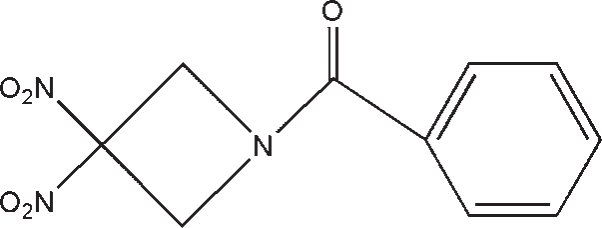


## Experimental

### 

#### Crystal data

C_10_H_9_N_3_O_5_
*M* *_r_* = 251.20
Monoclinic, 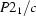

*a* = 13.176 (4) Å
*b* = 6.2344 (19) Å
*c* = 13.522 (4) Å
β = 92.612 (6)°
*V* = 1109.6 (6) Å^3^
*Z* = 4
Mo *K*α radiation
μ = 0.12 mm^−1^
*T* = 296 K
0.39 × 0.27 × 0.15 mm

#### Data collection

Bruker SMART APEXII diffractometer
Absorption correction: multi-scan (*SADABS*; Sheldrick, 2000 ▶) *T*_min_ = 0.954, *T*_max_ = 0.981
5306 measured reflections
1975 independent reflections
1210 reflections with *I* > 2σ(*I*)
*R*_int_ = 0.028

#### Refinement

*R*[*F* ^2^ > 2σ(*F* ^2^)] = 0.035
*wR*(*F* ^2^) = 0.096
*S* = 0.98
1975 reflections
164 parameters
H-atom parameters constrained
Δρ_max_ = 0.15 e Å^−3^
Δρ_min_ = −0.16 e Å^−3^

### 

Data collection: *SMART* (Bruker, 2003 ▶); cell refinement: *SAINT* (Bruker, 2003 ▶); data reduction: *SAINT*; program(s) used to solve structure: *SHELXS97* (Sheldrick, 2008 ▶); program(s) used to refine structure: *SHELXL97* (Sheldrick, 2008 ▶); molecular graphics: *SHELXTL* (Sheldrick, 2008 ▶); software used to prepare material for publication: *SHELXTL*.

## Supplementary Material

Crystal structure: contains datablocks I, global. DOI: 10.1107/S1600536809051290/gk2242sup1.cif

Structure factors: contains datablocks I. DOI: 10.1107/S1600536809051290/gk2242Isup2.hkl

Additional supplementary materials:  crystallographic information; 3D view; checkCIF report

## Figures and Tables

**Fig. 1.**
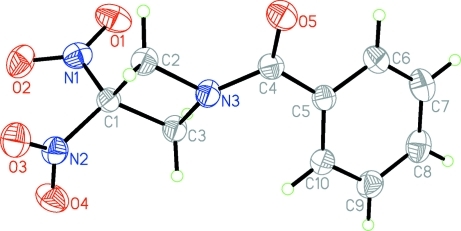
The molecular structure of the title compound showing the atom-numbering scheme. Displacement ellipsoids are drawn at the 30% probability level and H atoms are drawn as spheres of arbitrary radius.

## supplementary crystallographic information

### Comment

Dinitro- and trinitro-derivatives of azetidine are of interest because they contain strained ring system. This makes them good candidates for energetic materials (propellants or explosives). Initial reports on the synthesis of 1,3,3-trinitroazetidine (TNAZ) included the synthesis of 3,3-dinitroazetidine (DNAZ) in the synthesis pathway (Archibald *et al.*, 1990). However, later on less expensive synthesis of DNAZ was reported (Hiskey *et al.*, 1994*a*,*b*). Starting from DNAZ as a substrate a variety of solid energetic compounds can be prepared (Gao *et al.*, 2009; Ma, Yan, Li, Guan *et al.*, 2009; Ma, Yan, Li, Song & Hu, 2009; Ma, Yan, Song *et al.*, 2009). This paper reports synthesis and crystal structure of the title DNAZ derivate.

### Experimental

A solution of DNAZ (0.40 g, 2.72 mmol), benzoyl chloride (0.35 ml, 2.99 mmol) and NaHCO_3_ (0.23 g, 2.72 mmol) in dichloromethane (20.0 ml) was stirred under reflux for 16 h. The reaction mixture was concentrated *in vacuo*, acetone (30.0 ml) was added, and the mixture was stirred for 30 min, standing, filtered. The solid product was washed with ethanol and purified by recrystallization from dichloromethane to give the pure colorless compound in 81.7% yield. The title compound (52 mg,0.2 mmol) was dissolved in chloroform (10 ml). Colorless crystals were isolated after several days. Elemental analysis calculated for C_10_H_9_N_3_O_5_: C 47.81, N 16.73, H 3.61%; found: C 47.29, N 16.88, H 3.63%. IR (KBr, cm^-1^): 3057, 2961, 1640, 1578, 1526, 1335, 1304, 706. ^1^H NMR (CDCl_3_): (δdelta/p.p.m.) 7.649 (2*H*), 7.581 (4*H*), 7.489 (2*H*), 5.025 (4*H*).

### Refinement

All H atoms were placed at calculated idealized positions and refined using a riding model, with C—H distances in the range 0.93–0.97 Å.

### Crystal data

**Table:**

C_10_H_9_N_3_O_5_	*F*(000) = 520
*M**_r_* = 251.20	*D*_x_ = 1.504 Mg m^−^^3^
Monoclinic, *P*2_1_/*c*	Mo *K*α radiation, λ = 0.71073 Å
Hall symbol: -P 2ybc	Cell parameters from 862 reflections
*a* = 13.176 (4) Å	θ = 3.0–21.2°
*b* = 6.2344 (19) Å	µ = 0.12 mm^−^^1^
*c* = 13.522 (4) Å	*T* = 296 K
β = 92.612 (6)°	Block, colorless
*V* = 1109.6 (6) Å^3^	0.39 × 0.27 × 0.15 mm
*Z* = 4	

### Data collection

**Table:**

Bruker SMART APEXII diffractometer	1975 independent reflections
Radiation source: fine-focus sealed tube	1210 reflections with *I* > 2σ(*I*)
graphite	*R*_int_ = 0.028
phi and ω scans	θ_max_ = 25.1°, θ_min_ = 3.0°
Absorption correction: multi-scan (*SADABS*; Sheldrick, 2000)	*h* = −15→15
*T*_min_ = 0.954, *T*_max_ = 0.981	*k* = −7→7
5306 measured reflections	*l* = −15→11

### Refinement

**Table:**

Refinement on *F*^2^	Secondary atom site location: difference Fourier map
Least-squares matrix: full	Hydrogen site location: inferred from neighbouring sites
*R*[*F*^2^ > 2σ(*F*^2^)] = 0.035	H-atom parameters constrained
*wR*(*F*^2^) = 0.096	*w* = 1/[σ^2^(*F*_o_^2^) + (0.0493*P*)^2^] where *P* = (*F*_o_^2^ + 2*F*_c_^2^)/3
*S* = 0.98	(Δ/σ)_max_ = 0.001
1975 reflections	Δρ_max_ = 0.15 e Å^−^^3^
164 parameters	Δρ_min_ = −0.16 e Å^−^^3^
0 restraints	Extinction correction: *SHELXL*, Fc^*^=kFc[1+0.001xFc^2^λ^3^/sin(2θ)]^-1/4^
Primary atom site location: structure-invariant direct methods	Extinction coefficient: 0.014 (2)

### Special details

**Table:**

Geometry. All esds (except the esd in the dihedral angle between two l.s. planes) are estimated using the full covariance matrix. The cell esds are taken into account individually in the estimation of esds in distances, angles and torsion angles; correlations between esds in cell parameters are only used when they are defined by crystal symmetry. An approximate (isotropic) treatment of cell esds is used for estimating esds involving l.s. planes.
Refinement. Refinement of *F*^2^ against ALL reflections. The weighted *R*-factor *wR* and goodness of fit *S* are based on *F*^2^, conventional *R*-factors *R* are based on *F*, with *F* set to zero for negative *F*^2^. The threshold expression of *F*^2^ > σ(*F*^2^) is used only for calculating *R*-factors(gt) *etc*. and is not relevant to the choice of reflections for refinement. *R*-factors based on *F*^2^ are statistically about twice as large as those based on *F*, and *R*- factors based on ALL data will be even larger.

### Fractional atomic coordinates and isotropic or equivalent isotropic displacement parameters (Å^2^)

**Table:**

	*x*	*y*	*z*	*U*_iso_*/*U*_eq_	
N3	0.68948 (11)	0.3359 (2)	0.80874 (11)	0.0468 (4)	
O5	0.75063 (10)	0.01052 (19)	0.84162 (10)	0.0599 (4)	
O1	0.75527 (11)	0.6527 (2)	0.99733 (12)	0.0775 (5)	
O2	0.61765 (11)	0.8369 (2)	1.01338 (11)	0.0705 (5)	
O3	0.44932 (11)	0.6147 (2)	0.89476 (11)	0.0713 (5)	
O4	0.52323 (10)	0.8514 (2)	0.80573 (12)	0.0702 (5)	
C6	0.91631 (15)	0.0829 (3)	0.71825 (16)	0.0579 (6)	
H6	0.9293	−0.0078	0.7719	0.070*	
C7	0.98541 (16)	0.0983 (3)	0.64582 (19)	0.0692 (6)	
H7	1.0451	0.0188	0.6510	0.083*	
C8	0.96726 (16)	0.2296 (3)	0.56602 (18)	0.0654 (6)	
H8	1.0145	0.2398	0.5172	0.078*	
C9	0.87872 (16)	0.3464 (3)	0.55826 (16)	0.0601 (6)	
H9	0.8658	0.4351	0.5038	0.072*	
C10	0.80912 (15)	0.3321 (3)	0.63113 (14)	0.0509 (5)	
H10	0.7494	0.4115	0.6255	0.061*	
C5	0.82730 (13)	0.2012 (3)	0.71215 (14)	0.0439 (5)	
C4	0.75447 (13)	0.1743 (3)	0.79194 (14)	0.0444 (5)	
C3	0.69359 (15)	0.5702 (3)	0.79507 (14)	0.0498 (5)	
H3A	0.6629	0.6195	0.7326	0.060*	
H3B	0.7607	0.6314	0.8072	0.060*	
C1	0.62456 (13)	0.5904 (3)	0.88225 (13)	0.0419 (5)	
C2	0.62480 (14)	0.3459 (3)	0.89365 (14)	0.0489 (5)	
H2B	0.6575	0.2956	0.9551	0.059*	
H2A	0.5586	0.2801	0.8821	0.059*	
N1	0.66923 (14)	0.7057 (3)	0.97243 (13)	0.0541 (5)	
N2	0.52333 (12)	0.6935 (3)	0.85921 (13)	0.0513 (4)	

### Atomic displacement parameters (Å^2^)

**Table:**

	*U*^11^	*U*^22^	*U*^33^	*U*^12^	*U*^13^	*U*^23^
N3	0.0634 (10)	0.0315 (8)	0.0468 (10)	0.0014 (7)	0.0156 (8)	0.0040 (7)
O5	0.0819 (10)	0.0343 (7)	0.0641 (9)	0.0015 (6)	0.0103 (8)	0.0088 (7)
O1	0.0730 (10)	0.0680 (10)	0.0889 (13)	−0.0016 (8)	−0.0258 (9)	0.0058 (8)
O2	0.0914 (11)	0.0603 (9)	0.0607 (10)	−0.0037 (8)	0.0135 (9)	−0.0178 (8)
O3	0.0565 (9)	0.0876 (12)	0.0710 (11)	0.0034 (8)	0.0172 (8)	0.0072 (9)
O4	0.0717 (10)	0.0584 (9)	0.0800 (11)	0.0119 (7)	−0.0017 (8)	0.0189 (8)
C6	0.0657 (13)	0.0471 (12)	0.0611 (14)	0.0105 (10)	0.0041 (12)	0.0029 (10)
C7	0.0581 (13)	0.0652 (14)	0.0848 (18)	0.0108 (11)	0.0108 (13)	−0.0071 (13)
C8	0.0668 (14)	0.0598 (13)	0.0712 (17)	−0.0057 (11)	0.0222 (12)	−0.0085 (12)
C9	0.0764 (14)	0.0533 (13)	0.0513 (13)	0.0010 (11)	0.0115 (11)	0.0033 (10)
C10	0.0579 (11)	0.0497 (12)	0.0453 (12)	0.0058 (9)	0.0050 (10)	−0.0012 (10)
C5	0.0536 (11)	0.0324 (10)	0.0454 (12)	−0.0006 (8)	0.0004 (9)	−0.0043 (9)
C4	0.0553 (11)	0.0320 (10)	0.0454 (11)	−0.0013 (9)	−0.0008 (9)	−0.0019 (9)
C3	0.0641 (12)	0.0355 (10)	0.0511 (12)	0.0046 (8)	0.0147 (10)	0.0054 (9)
C1	0.0495 (11)	0.0356 (9)	0.0407 (11)	0.0024 (8)	0.0038 (9)	0.0001 (8)
C2	0.0604 (11)	0.0393 (10)	0.0477 (12)	−0.0017 (9)	0.0101 (9)	0.0022 (9)
N1	0.0679 (12)	0.0413 (10)	0.0528 (11)	−0.0070 (9)	0.0002 (10)	0.0038 (8)
N2	0.0565 (11)	0.0492 (10)	0.0482 (10)	0.0039 (9)	0.0035 (8)	−0.0039 (8)

### Geometric parameters (Å, °)

**Table:**

N3—C4	1.348 (2)	C8—H8	0.9300
N3—C2	1.462 (2)	C9—C10	1.379 (3)
N3—C3	1.474 (2)	C9—H9	0.9300
O5—C4	1.224 (2)	C10—C5	1.378 (3)
O1—N1	1.2134 (19)	C10—H10	0.9300
O2—N1	1.2133 (18)	C5—C4	1.486 (2)
O3—N2	1.2109 (19)	C3—C1	1.527 (2)
O4—N2	1.2212 (19)	C3—H3A	0.9700
C6—C7	1.371 (3)	C3—H3B	0.9700
C6—C5	1.385 (2)	C1—N2	1.500 (2)
C6—H6	0.9300	C1—N1	1.511 (2)
C7—C8	1.367 (3)	C1—C2	1.532 (2)
C7—H7	0.9300	C2—H2B	0.9700
C8—C9	1.375 (3)	C2—H2A	0.9700
			
C4—N3—C2	124.17 (15)	N3—C3—C1	87.62 (12)
C4—N3—C3	133.82 (14)	N3—C3—H3A	114.0
C2—N3—C3	94.70 (12)	C1—C3—H3A	114.0
C7—C6—C5	120.6 (2)	N3—C3—H3B	114.0
C7—C6—H6	119.7	C1—C3—H3B	114.0
C5—C6—H6	119.7	H3A—C3—H3B	111.2
C8—C7—C6	120.5 (2)	N2—C1—N1	105.88 (14)
C8—C7—H7	119.7	N2—C1—C3	115.48 (15)
C6—C7—H7	119.7	N1—C1—C3	116.01 (15)
C7—C8—C9	119.7 (2)	N2—C1—C2	116.42 (14)
C7—C8—H8	120.2	N1—C1—C2	113.13 (15)
C9—C8—H8	120.2	C3—C1—C2	89.82 (12)
C8—C9—C10	120.0 (2)	N3—C2—C1	87.84 (12)
C8—C9—H9	120.0	N3—C2—H2B	114.0
C10—C9—H9	120.0	C1—C2—H2B	114.0
C5—C10—C9	120.60 (18)	N3—C2—H2A	114.0
C5—C10—H10	119.7	C1—C2—H2A	114.0
C9—C10—H10	119.7	H2B—C2—H2A	111.2
C10—C5—C6	118.61 (18)	O2—N1—O1	126.33 (18)
C10—C5—C4	123.34 (16)	O2—N1—C1	118.88 (17)
C6—C5—C4	118.01 (18)	O1—N1—C1	114.78 (17)
O5—C4—N3	119.23 (17)	O3—N2—O4	125.66 (17)
O5—C4—C5	122.48 (16)	O3—N2—C1	117.90 (16)
N3—C4—C5	118.29 (15)	O4—N2—C1	116.44 (16)
			
C5—C6—C7—C8	−0.5 (3)	N3—C3—C1—N1	116.69 (15)
C6—C7—C8—C9	−0.3 (3)	N3—C3—C1—C2	0.88 (14)
C7—C8—C9—C10	0.5 (3)	C4—N3—C2—C1	154.51 (16)
C8—C9—C10—C5	0.0 (3)	C3—N3—C2—C1	0.92 (14)
C9—C10—C5—C6	−0.7 (3)	N2—C1—C2—N3	117.73 (15)
C9—C10—C5—C4	−178.42 (16)	N1—C1—C2—N3	−119.27 (16)
C7—C6—C5—C10	0.9 (3)	C3—C1—C2—N3	−0.88 (14)
C7—C6—C5—C4	178.79 (17)	N2—C1—N1—O2	6.0 (2)
C2—N3—C4—O5	10.7 (3)	C3—C1—N1—O2	135.59 (16)
C3—N3—C4—O5	152.76 (19)	C2—C1—N1—O2	−122.61 (16)
C2—N3—C4—C5	−170.27 (16)	N2—C1—N1—O1	−174.84 (15)
C3—N3—C4—C5	−28.2 (3)	C3—C1—N1—O1	−45.3 (2)
C10—C5—C4—O5	152.40 (18)	C2—C1—N1—O1	56.5 (2)
C6—C5—C4—O5	−25.3 (3)	N1—C1—N2—O3	−92.40 (18)
C10—C5—C4—N3	−26.6 (2)	C3—C1—N2—O3	137.76 (16)
C6—C5—C4—N3	155.63 (16)	C2—C1—N2—O3	34.3 (2)
C4—N3—C3—C1	−150.25 (19)	N1—C1—N2—O4	87.52 (18)
C2—N3—C3—C1	−0.92 (14)	C3—C1—N2—O4	−42.3 (2)
N3—C3—C1—N2	−118.56 (15)	C2—C1—N2—O4	−145.79 (16)
